# Fibrous tumor of the stomach causing abdominal pain and melena: Case report with review of literature

**DOI:** 10.1016/j.ijscr.2019.10.045

**Published:** 2019-10-31

**Authors:** Wan Aldohuky, Ayad Ahmad Mohammed

**Affiliations:** a Department of Surgery, Duhok Directorate General of Health, Kurdistan Region, Iraq; b Department of Surgery, College of Medicine, University of Duhok, Kurdistan Region, Iraq

**Keywords:** Fibrous tumors, Dystrophic calcifications, Abdominal pain, Gastric tumors, Endoscopy

## Abstract

**Introduction:**

Fibrous tumor of the stomach is a rare mesenchymal tumor composed of hyalinized fibrous tissue with spindle shaped fibroblasts. This type of tumor usually occurs in the subcutaneous tissues and the deep soft tissues. Its occurrence in stomach is extremely a rare finding and only few cases have been reported worldwide, the exact etiology is still unclear.

**Presentation of case:**

A 68-year-old female was complaining from epigastric and right hypochondrial pain for few months. It was associated with attacks of non-bilious vomiting and early satiety, with attacks of melena. During abdominal examination there was a palpable mass in the epigastric region which was oval in shape, soft, mobile in the vertical direction, and was non-pulsatile. The hemoglobin was 9 gm/liter. Ultrasound and CT-scan of the abdomen showed a well-defined non-enhancing, multi-loculated mass related to the body of the stomach. Gastroscopy showed soft tissue mass with intact mucosa in the anterior gastric wall, multiple biopsies were taken which revealed fatty tissue with no evidence of malignancy.

Laparotomy was done and a soft, mobile mass was found in the anterior gastric wall in the pre-pyloric region, the serosa was intact, complete excision of the mass was performed.

The histopathological result showed fibrous tumors of the stomach.

**Conclusion:**

The treatment of this tumor is mainly through surgical intervention. Which should be done at the time of diagnosis. The prognosis after successful resection is excellent, few cases are reported with local recurrence.

## Highlights

• Fibrous tumors are rare mesenchymal tumors composed of hyalinized fibrous tissue with spindle shaped fibroblasts.
• The treatment of these tumors is mainly through surgical intervention.
• The prognosis of these tumor after successful resection is excellent.

## 1 Introduction

Fibrous tumor of the stomach is a rare type of benign mesenchymal tumors composed of hyalinized fibrous tissue with spindle shaped fibroblasts, the tumor may contain areas of dystrophic calcifications and variable degree of mononuclear inflammatory cell infiltration. This tumor usually occurs in the subcutaneous tissues, the deep soft tissues, the subserosal tissues, and the gastrointestinal tract. Its occurrence in the gastric wall is an extremely rare finding and only few cases have been reported worldwide, the exact etiology is still unclear [1,2].

The tumor may be completely asymptomatic or may cause a variety of symptoms depending on its anatomical location. Gastric involvement may asymptomatic, or may present with epigastric pain, nausea, vomiting which may be bloody due to mucosal erosion, melena, iron deficiency anemia, pyloric obstruction. The characteristic histopathological feature is the presence of spindle cells that express factor XIIIa [2].

The CT-scan findings of these tumors typically show well delineated outlines with lobulated appearance, occasionally there may be scattered calcifications inside the tumor [3].

The cut surface of the tumor is pale brown or pale yellow in color, with cystic changes, the tumor may also contain stony hard nodules due to focal areas of calcifications [4].

The biological behavior of the tumors located in the abdominal viscera is not clearly studied due to rarity of the tumor, and many cases are discovered on autopsy samples [2].

Surgery is the principal option of treatment which can be done endoscopically, or by surgical resection [5].

The work of this case report has been reported in line with the SCARE 2018 criteria [6].

## 2 Patient information

A 68-year-old female was complaining from epigastric and right hypochondrial pain for few months, the pain was dull aching in type and poorly localized, coming in attacks and associated with meals, there was no radiation of the pain. There patient had no nausea or vomiting. The patient visited the hospital and the initial investigations and ultrasound of the abdomen showed multiple gall stones with no signs of inflammation. The pain was attributed to gallstones. The patient had diabetes mellitus which was controlled with oral antidiabetic drugs and dietary modifications, the past surgical history was negative.

Laparoscopic cholecystectomy was done for the patient and the patient was discharged home after 2 days. One year after cholecystectomy the patient presented with epigastric pain which was increasing in intensity, and was associated with attacks of non-bilious vomiting and early satiety. The patient had 2 attacks of melena. The patient had no history of weight loss.

The patient had a negative history for chronic drugs administration other than the oral hypoglycemic drugs. There was no family history for similar problems or for genetic abnormalities. The psychosocial history was non-relevant.

### 2.1 Clinical findings

During abdominal examination there was a palpable mass in the epigastric region which was oval in shape, soft, mobile in the vertical direction, and was non-pulsatile. The patient had normal pulse, blood pressure, and temperature.

The hemoglobin level was 9 gm/liter and the white blood cells and the inflammatory markers were normal.

### 2.2 Diagnostic assessment

**Fig. 1:**
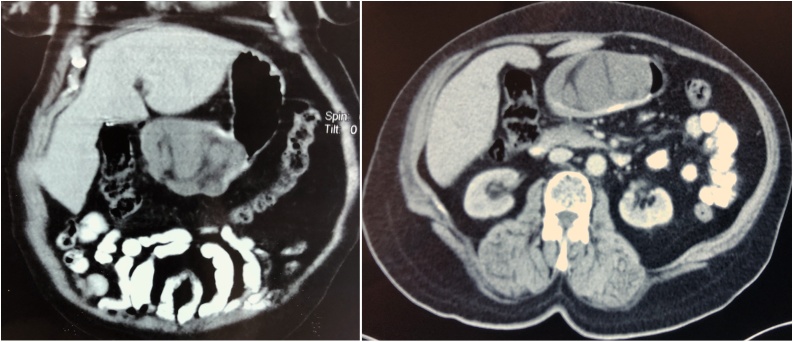
CT-scan of the abdomen showing evidence of a large non-enhancing intramural mass in the gastric wall with narrowing of the pyloric canal.

Abdominal ultrasound and CT-scan of the abdomen showed a well-defined non-enhancing, multi-loculated lesion measuring 9 × 4 cm related to the body of the stomach, the lesion contained mixed fatty and soft tissue components (Fig. 1).

**Fig. 2:**
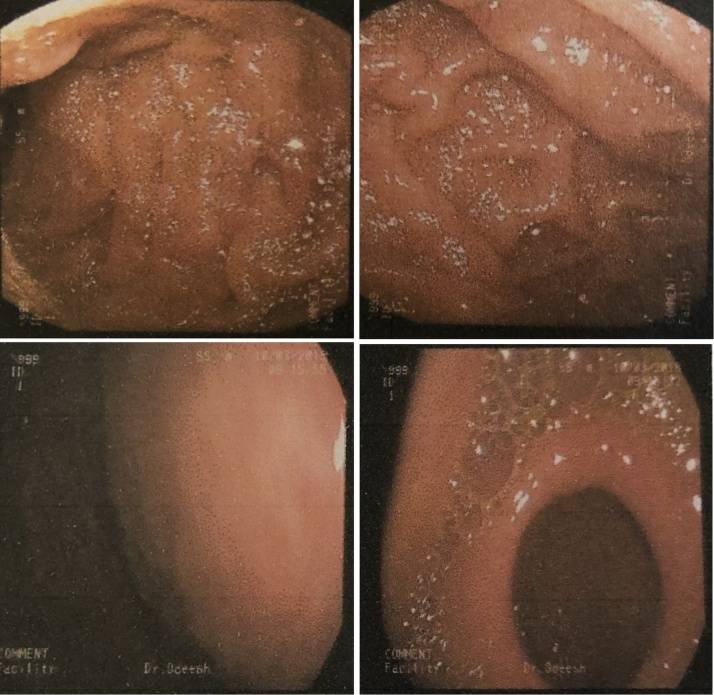
An endoscopic view of the stomach showing a soft tissue mass causing bulging of the gastric wall with intact mucosa over it.

Gastroscopy showed a soft tissue mass with an intact mucosa in the anterior gastric wall, biopsies were taken which revealed fatty tissue with no evidence of malignancy (Fig. 2).

### 2.3 Therapeutic intervention

**Fig. 3:**
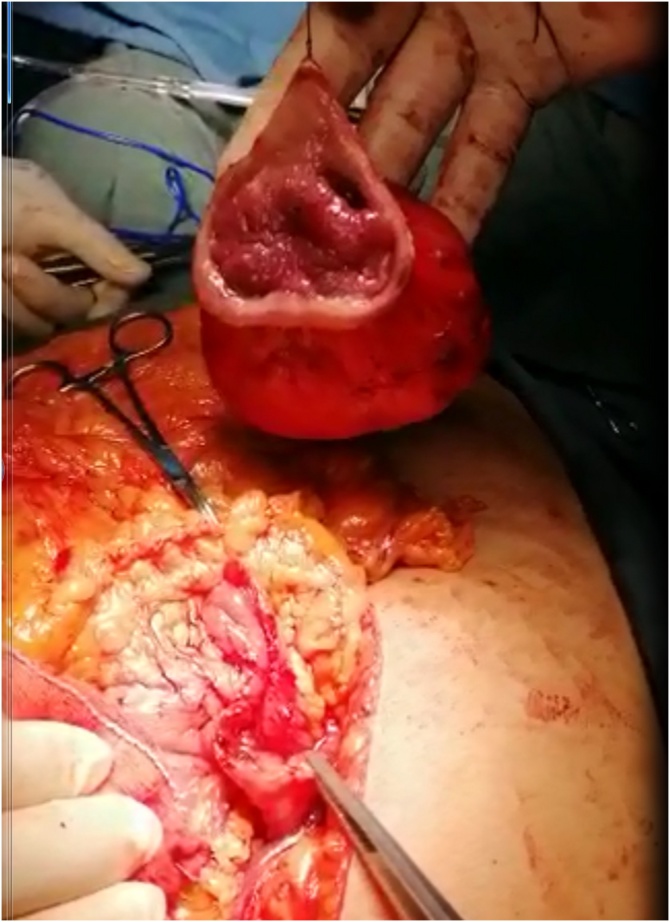
An intraoperative picture showing complete excision of the gastric mass.

Laparotomy was done through an upper midline incision, during surgery there was a soft, mobile mass which was related to the anterior gastric wall in the pre-pyloric region, the serosa was intact and there were no other masses in other parts of the bowel, and other organs (Fig. 3).

**Fig. 4:**
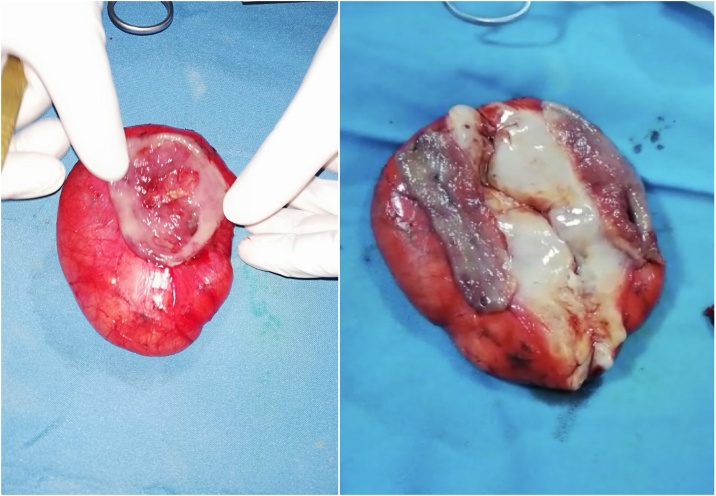
An intraoperative picture showing the cut surface of the mass after excision, the inner surface of the mass is whitish-yellow and the consistency is soft.

Excision of the mass was performed with a rim of 2 cm apparently healthy tissue; the mass was causing mucosal ulceration. The gastric wall was closed in 2 layers using a slowly absorbable suture material (Fig. 4).

**Fig. 5:**
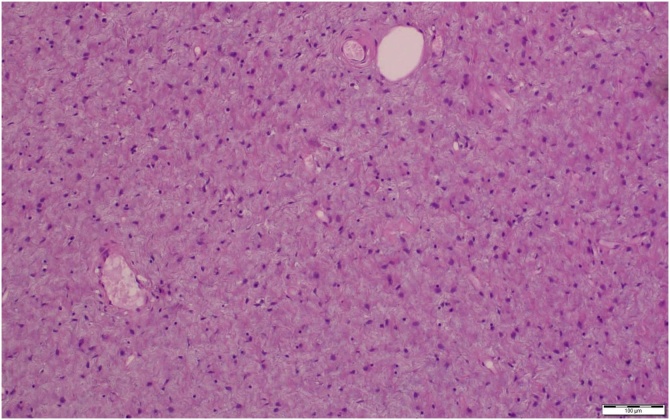
A microscopic picture showing a hypo-cellular myxoid tissue with ramifying blood vessels, there are proliferating spindle cells with keloid like collagen bundles, no atypia or mitosis is detected.

The histopathological examination of the mass was containing hypo-cellular myxoid areas with small blood vessels, spindle cell and keloid like pattern of collagen, no atypia or mitosis were detected. The tumor was strongly positive for vimentin and CD34, and negative for desmin, H-caldesmon, SMA, S-100 protein. These features were consistent with fibrous tumors of the stomach arising from the subserosal tissue plane (Fig. 5).

The operation was performed by a general surgeon who is specialized in the field of gastrointestinal surgery.

There were no any specific post-intervention considerations, but the patient was advised to avoid heavy meals for 2 months.

### 2.4 Follow-up and outcomes

The patient was admitted in the hospital for 5 days, and was discharged with good general clinical conditions with no postoperative complications.

## 3 Discussion

Solitary fibrous tumors are spindle cell tumors that predominantly occur in the pleura or the peritoneum, their occurrence in the stomach is an extremely rare finding. When these tumors arise from the pleura there are easily recognized but when arise in other anatomical locations, the diagnosis is very challenging [3].

The tumor can affect any age group and in a literature review for the reported cases, the mean size of the tumor was around 3 cm and there was male predominance, all tumors were unifocal, and occurring in old ages [1,4].

The etiology of such tumors is not well studied because they are uncommon and no adequate data are available, but when the tumors occur in the pediatric age group they are usually attributed to an embryonic insult, genetic derangements or vascular insults [4].

The differential diagnoses of such tumors include GISTs, leiomyoma, leiomyosarcoma, schwannoma, inflammatory fibrous polyps, desmoid tumors, inflammatory myoblastic tumor, plexiform fibromyxoma, and lipoma. The most important point is the differentiation of these tumors from gastrointestinal stromal tumors (GISTs), this can be done according to the differences in the attenuation during imaging, GISTs usually have a central lower attenuation due to central necrosis or hemorrhage, are relatively larger in size, have homogenous appearance, and have an exophytic growth when compared to fibrous tumors. The immunohistochemical studies and histopathological examination are the definitive tests for the differentiation [3,4,7].

The treatment of these tumors is mainly through surgical intervention. Surgical excision should be done at the time of diagnosis, surgical resection can be done adopting the open or the laparoscopic techniques, small size tumors are feasible for endoscopic resection. The prognosis of these tumor after successful resection is excellent in about 90% of the patients and long term follow up is not recommended in the majority of patients [5].

The learning message in this case report is that although gall stones are commonly diagnosed, but many patients are non-symptomatic, the patient’s symptoms should be evaluated carefully and the character of the pain analyzed before attributing them to gall stones. Patients may present with more than one pathology at the same time.

Patient’s perspective: I had the same attacks of pain before cholecystectomy and after it, I knew that there something going wrong in my abdomen until finally the doctors discovered that the problem is in my stomach.

## Conflicts of interest

The author has no conflicts of interest to declare.

## Sources of funding

None.

## Ethical approval

Ethical approval has been exempted by my institution for reporting this case.

## Consent

Written informed consent was obtained from the patient for publication of this case report and accompanying images.

## Author contribution

The concept of reporting the case, data recording, and drafting the work done by Dr Wan Aldohuky and Dr Ayad Ahmad Mohammed.

Dr Wan Aldohuky took the consent from the patient for publishing the case.

Final approval of the work to be published was done by Dr Ayad Ahmad Mohammed.

## Registration of research studies

This work is case report and there is no need of registration.

## Guarantor

Dr Ayad Ahmad Mohammed is guarantor for the work.

## Provenance and peer review

Not commissioned, externally peer-reviewed.
